# Heads-Up 3D Surgery under Low Light Intensity Conditions: New High-Sensitivity HD Camera for Ophthalmological Microscopes

**DOI:** 10.1155/2019/5013463

**Published:** 2019-11-25

**Authors:** Celso Soiti Matsumoto, Masayuki Shibuya, Jun Makita, Takuhei Shoji, Hisato Ohno, Kei Shinoda, Harue Matsumoto

**Affiliations:** ^1^Department of Ophthalmology, Teikyo University School of Medicine, Kaga 2-11-1, Itabashi-ku, Tokyo 173-8605, Japan; ^2^Department of Ophthalmology, Saitama Medical University School of Medicine, Morohongo 38, Moroyama-machi, Saitama 350-0495, Japan; ^3^Matsumoto Eye Clinic, Tokushima, Japan; ^4^Ohno Eye Clinic, Saitama, Japan

## Abstract

**Purpose:**

To determine the feasibility of performing intraocular surgeries in a heads-up position with low illuminance conditions by observing a display of the surgical field created by a three-dimensional imaging (3D) system.

**Methods:**

Seventy-four eyes of 56 patients underwent cataract surgery (72 eyes) with the heads-up 3D surgery system; 60 eyes with cataract surgery alone, 7 eyes with combined cataract and glaucoma microdevice implant surgery, 5 eyes with combined cataract and vitrectomy surgery, and two eyes with vitrectomy surgery alone were studied. The illuminance from the surgical microscope was set to be dimmer (Leica M822F40 main light 2%; otto-flex 6%) than the usual setting to minimize the discomfort and glare for the patient. The surgeries were performed under topical anesthesia. The luminance of the images observed through the eyepieces of the operating microscope and the image of a 3D system created by a high-sensitivity sensor Exmor R 3CMOS HD camera (Sony MCC-1000MD) were measured.

**Results:**

All surgeries were completed without any complications under the low illumination conditions. The surgical field on the display monitor was created by a 3D system using a high-sensitivity sensor camera and was observed in a heads-up position. The patients did not report any intolerable discomfort or glare during the surgery. Cataract surgeries were performed with a good view of the surgical field under the extremely low illumination from the surgical microscope. The high-sensitivity sensors and electronic amplifications of the image signals made the surgical field brighter and allowed the surgeon to perform the surgery confidently and safely.

**Conclusions:**

Heads-up, 3D-assisted intraocular surgeries can be performed safely and efficiently with low illuminance of the surgical field. This trial is registered with UMIN000037838.

## 1. Introduction

Earlier studies have reported that the “heads-up surgery” method was well suited for cataract and vitreoretinal surgery, and it is expected that this method will become more widely used with further technical improvements [1–3]. The term heads-up surgery was introduced in earlier clinical studies, and it meant that the surgeon performs surgery by viewing the image of the surgical field created on a three-dimensional display screen in a heads-up position and not by looking down through the microscope eyepieces. Eckardt and Paulo reported that the heads-up surgery method had better ergonomics than the traditional heads-down method and led to less excessive neck and body fatigue for the surgeon [3]. Heads-up surgery also allowed the surgical staff and residents to observe the same image seen by the surgeon and to learn from this setup.

We have used the terms, “digital-assisted surgery (DAS)” and “digitalized heads-up surgery (DHS)” to describe the surgical methods that were used in this study. This technique used digital microscopes for the surgeries even though analogue microscopes have higher resolution.

Surgical microscopes for ophthalmology have been improved in many ways, e.g., the use of 0-degree light beams to reduce the level of retroillumination, static laser filters, navigation systems for cataract surgeries, intraoperative OCT recordings and viewing, and laser filters in the microscope optical pathway. These alterations will result in a significant reduction in the brightness of the surgical field even though surgeons prefer to increase the luminance of the surgical field. As a result, the illuminance of the operative field can be excessively bright which can be harmful to the retina and cause discomfort to the patients. In contrast, DAS with extremely high-sensitivity cameras can amplify the image signals and allow a brighter surgical field for surgeons in low luminance conditions of the microscope light.

The purpose of this study was to determine the efficacy of using a new, highly sensitive HD camera with high dynamic range (HDR) to create 3D images of the surgical field on a monitor to perform heads-up surgery. This study evaluated whether this system was as effective and safe as the traditional surgeries with an operating microscope [3].

## 2. Materials and Methods

### 2.1. Equipment

#### 2.1.1. Experimental Equipment Setup

An operating microscope (model M822®, Leica Microsystems, Wetzlar, Germany) combined with a pair of HD camera synchronized to each other was used to create the 3D images. The image signals were digitalized by the video camera controller unit (MCC-1000MD, Sony Corporation, Tokyo, Japan) which operates with the latest generation image sensor technology (Exmor R, Sony Corporation, Tokyo Japan). This combination allowed better sensitivity at low-light levels than the commercial cameras.

The recorded HD images were converted to 3D images by a medically used 3D 4 K LCD display (model LMD-X550MT, Sony Corporation, Tokyo Japan). To observe the surgical field in 3D, the surgeon wears polarized glasses. The distance from the 55-inch LCD monitor to the surgeon is approximately 300 cm, and the viewable image size was 68.45 cm × 121.54 cm (height × width).

To obtain images with good resolution and good depth of field, the iris aperture of the video camera adapter was reduced to 9 mm in diameter which is 60% of the fully opened diameter (15 mm). The video camera settings were resolution, 1920 × 1080/60 pixels; picture exposure mode, auto; high-sensitivity mode, off; shutter speed, auto (max 1 : 2000); and brightness, default setting.

**Figure 1:**
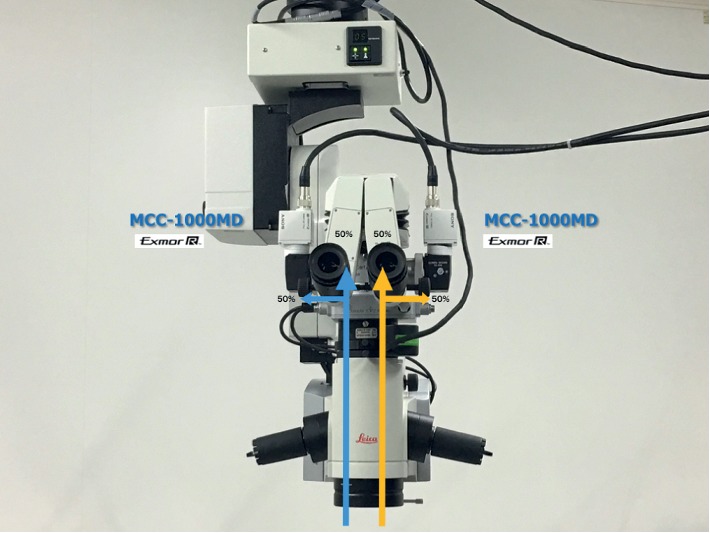
A beam splitter divides into half the light beam was placed at the microscope optics for direct observation and for the 3D digital system.

A 50/50 beam splitter (Leica, Wetzlar, Germany) that divided the viewing light beam into two was placed in the microscope optical path so that one beam was used for direct observation and the other beam for the 3D digital system (Figure 1).

For experimental measures, the main light of the microscope was set at an intensity of 2% of the maximum for surgical field illumination. The microscope's second retroillumination halogen light source (0-degree light beam or otto-flex light) was not used for this study for experimental measurements.

#### 2.1.2. Brightness Measurements of Images from Eyepieces and from 3D Display

**Figure 2:**
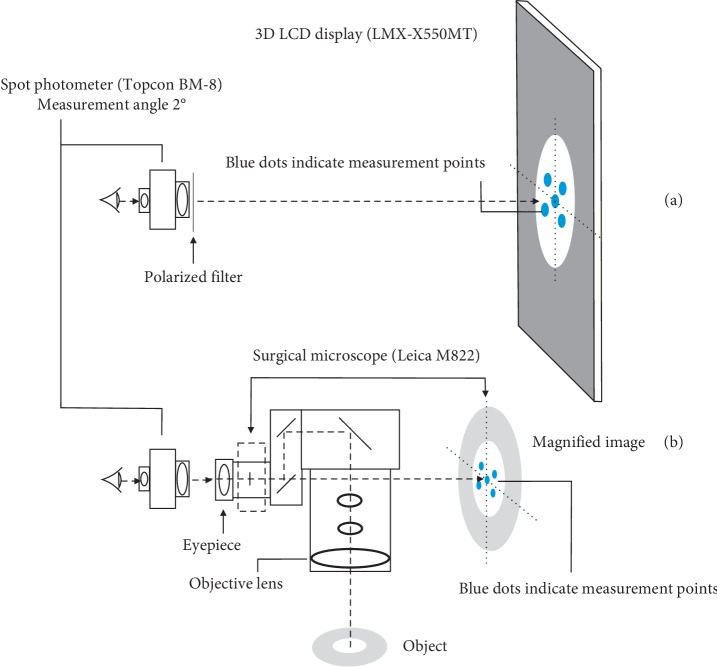
Schematic figure of the measurement of the luminance of the images produced by a 3D LCD display (a) and by an analogue microscopic view (b). The measurement points are indicates by blue dots on the display and on the image obtained through the eyepiece.

The luminous emittance (lux) of the microscope light was measured at 200 mm from the objective lens by a luminance meter (Topcon, IM-3, Tokyo, Japan). The measurements of the luminance of the image obtained through the eyepiece and the image projected onto the 3D monitor screen were measured using a spot photometer (Topcon, BM-8, Tokyo, Japan) (Figure 2). The spot photometer measured a circular 2° area in the visual field, and the measurements were made at five locations; one at the center and one in each of the 4 quadrants of the images. The results were averaged to minimize the effect of differences in the brightness at the different locations.

The microscope's main light was set at 2% of its maximum, and it was focused and projected onto a white reflectance surface. The microscope magnification was set at 5X.

#### 2.1.3. Equipment Setup for Clinical Use

The same microscope and imaging system were used for clinical measurements.

The main halogen light (2-degree light source) of the microscope was used as the light source. For clinical applications, the main light of the microscope was set at an intensity of 2% of the maximum for surgical field illumination. The microscope's second retroillumination halogen light source (0-degree light beam or otto-flex light) was set at 6% for clinical applications. The intensities of the light source preset value areas are defined below.

The microscope light sources were preset to not cause excessive discomfort and glare for the patient during the surgery. Ten young volunteers had their right pupils dilated with topical tropicamide (0.5%) 30 minutes prior to the exposure of the operating microscope light. The preset microscopic illumination was used, and the subjects were asked at what intensity they felt uncomfortable by the light exposure. The answers were averaged. A dazzling sensation was positive for 4% for the main light and 9% for the otto-flex light. We concluded that the otto-flex light was the threshold for a dazzling sensation. We decided to use the preset value below the threshold as 2% for main light and 6% for otto-flex retroillumination lights.

### 2.2. Clinical Study Using Low Illumination Heads-Up Surgery

Seventy-four eyes of 56 consecutive patients who were scheduled to undergo intraocular surgery were studied. There were 20 men and 36 women whose mean age was 75 ± 8.34 years (±standard deviation) with a range of 58 to 92 years. There were 72 eyes with senile cataract, 5 eyes with open angle glaucoma, 2 eyes with normal tension glaucoma, 5 eyes with epiretinal membrane, and 2 eyes with vitreous opacity.

The procedures used conformed to the tenets of the Declaration of Helsinki, and they were approved by the Review Board of Matsumoto Eye Clinic. An informed consent was obtained from all patients after an explanation of the purpose of the study, procedures to be used, and possible complications. Soon after the end of the surgery, the patients were questioned about their comfort and dazzling illumination during the surgery.

#### 2.2.1. Surgical Procedures

The surgeries were performed during February and March 2019, and all were performed by one of the authors (CSM) using the digital heads-up method. The eyes were anesthetized by topical oxybuprocaine hydrochloride (0.4%) and lidocaine (2%), and the pupils were dilated with topical tropicamide (0.5%) and phenylephrine hydrochloride (5%). The nonoperated eye was covered with the surgical drape. For vitrectomy, anesthesia was induced by a subtennon injection of lidocaine (2%) after the end of the cataract surgery. A capsular intrabag intraocular lens (IOL) was implanted in all of the cataract surgeries and combined phaco and vitrectomies or glaucoma surgeries. For the combined procedures, a standardized 3 port pars plana vitrectomy was performed with a 27-gauge vitrectomy system. The indications for vitrectomy included vitreous opacity due to chronic posterior uveitis without proliferative vitreoretinopathy and proliferative diabetic retinopathy, and prior macular surgeries including two cases of the macular epiretinal membrane.

All vitrectomy surgeries were performed with the 27-gauge port system, and the endoillumination levels were initially set to 3% of the maximum output. It was decreased to 2% for the epimacular membrane and ILM peeling procedures. The wide-field spread chandelier light was set to 3% of the maximum level.

During the study period, several other procedures, such as six glaucoma microdevice implantations (iStent, Glaukos) and seven vitrectomies (five vitrectomies were combined with cataract surgery), were performed using the heads-up technique. All operations were performed using the Constellation Vision System (Alcon Laboratories, Fort Worth, USA).

## 3. Results

### 3.1. Experimental Brightness Measurements of Images from Eyepieces and from 3D Display

**Figure 3:**
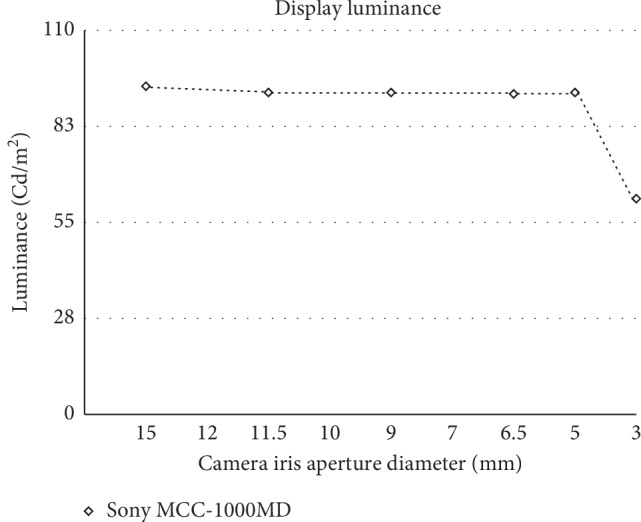
The highly sensitive HD video camera keeps capturing images with minimal loss in brightness when the iris aperture is narrowed to enlarge the depth of focus.

The luminance of the image at the eyepiece was 4 cd/m^2^. The 3D display luminance was measured at several iris aperture settings of the video camera (SD-033, Scimen Design, Tokyo, Japan). The image on the 3D display monitor was in average 92.2 cd/m^2^ (ranging 91.8 to 94 cd/m^2^) and was more than 23 times brighter than the image at the eyepiece (4 cd/m^2^) and 15 times brighter (61 cd/m^2^) even at the minimal diameter of the iris aperture. The highly sensitive HD video camera recorded the images with minimal loss in brightness when the iris aperture was reduced to increase the depth of focus (Figure 3).

### 3.2. Clinical Results Using Low-Illumination Heads-Up Surgery

**Figure 4:**
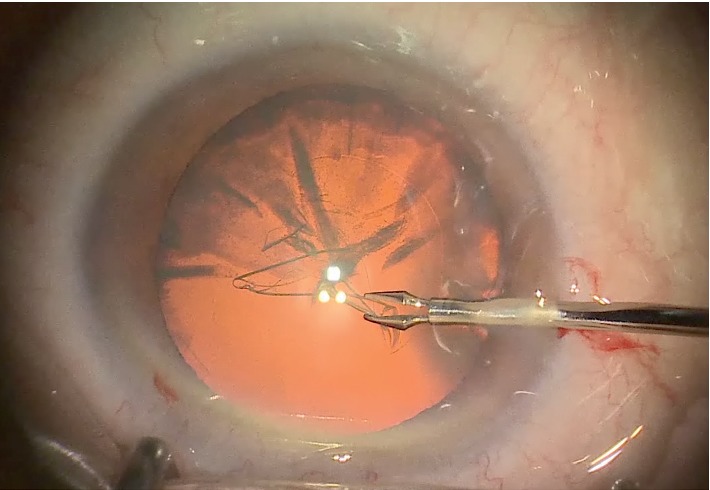
The visual field was clearly visualized with preset dim light during cataract surgery.

The view from the eyepieces under low illumination was too dim to perform surgery safely. So, only the 3D digitalized system was used for these surgeries. The visual field was clearly visible in the monitor in many cases with the preset dim light using the highly sensitive CMOS sensor video camera for 3D viewing (Figure 4). All cataract and glaucoma surgeries were performed with a 2% main light output (322 lux) and an otto-flex retroillumination light output of 6% (479 lux). However, the retroillumination light source brightness was increased from 6% to 7-8% (609–771 lux) of the maximum brightness to improve the view during the phacoemulsification procedures in five cases.

**Figure 5:**
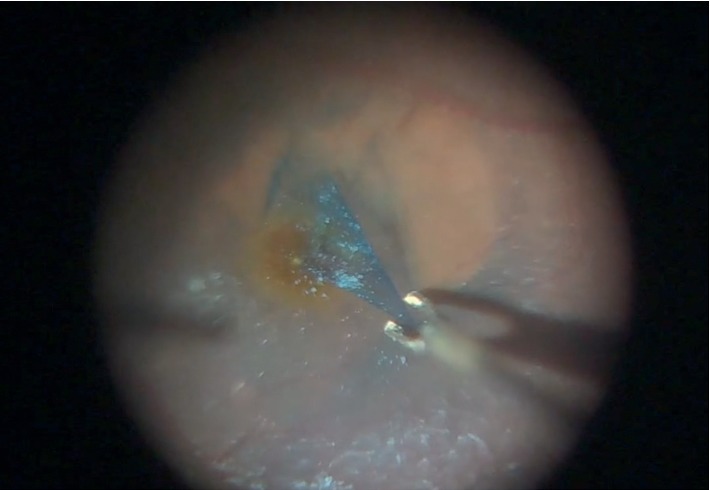
Good visualization of the macula during ILM peeling with a low endoillumination intensity of 2% (Constellation Vision System light source).

The endoillumination intensity for the 27-gauge port system during vitrectomy was 3% of the maximum output, and it was reduced to 2% for the epimacular membrane and ILM peeling procedures (Figure 5). The wide-field spread chandelier light was set to 3%.

No intra- or postoperative complications were observed in all cases. All patients were asked intraoperatively and just after the surgery whether the level of illumination was uncomfortable, and none answered that they were uncomfortable or that the light level was excessive.

## 4. Discussion

Heads-up, 3D digital surgery has many advantages over the conventional method including the increase of depth of focus, digital amplification of the image signal, better ergonomics for the surgeons, and live 3D view for all operation room staff. In addition, it allows the members of the medical team to see the surgical field without a reduction of the quality of view of the surgical field. Eckardt and Paulo reported that the current 2 K resolution, 3D heads-up surgical method was two times lower in resolution than that through the eyepieces [3]. Yamashita et al. developed an ultrahigh definition 8 K camera for surgical microscopes that is 16 times higher in resolution than the HD camera. They stated that the 8 K images were equal to the real microscopic images observed through the eyepieces [4]. However, increasing the resolution of the CMOS sensors has some limitations and disadvantages such as manufacturing it in small sizes is difficult and the need of more light to produce a clear bright image with high signal/noise ratio. There is also a decrease in the depth of focus thereby focusing becomes more difficult. In fact, commercial HD cameras have less definition than microscopic observation through the eyepieces. However, in practice, this difference is not noted during surgery and appears not to be a factor that compromises the surgery quality [3]. In the results of Eckardt and our experience, the resolution of the HD camera using 4 K display images seemed to be equal or might be better than the microscopic observation through the eyepieces. One reason could be that the video imaging process enhances the contrast and other parameters to provide a better view of the surgical field despite lower imaging resolution. Furthermore, Eckardt used questionnaires that were answered by surgeons comparing heads-up and traditional microscopic surgeries with convincing results that heads-up surgery had better results. They concluded that the electronic amplification of the camera imaging signals was useful in brightening the surgical field on the display monitor [3].

Our results showed that by using the Exmor R CMOS high-sensitive sensor video camera combined with Sony 4 K 3D monitor for 3D imaging, the luminance of the real image at the eyepiece was 23 times darker than that of the image obtained by 3D display in this system. The highly sensitive HD video camera records images with minimal loss in brightness even when the iris aperture is constricted to increase the depth of focus (Figure 3). The system was designed to deliver a high-level of details for microsurgery applications (https://pro.sony/ue_US/products/medical-cameras/mcc-1000md). The camera head integrates three Exmor R CMOS sensors. The sensors are combined with a refined image-processing technology that produces the sensitivity of F20, a signal-to-noise ratio of 63 dB. Simultaneous video outputs from two units can be synchronized, allowing recording of HD 3D stereoscopic video images. The high sensitivity of F20 means that this camera can record clear video images even in near darkness.

This system is especially useful for procedures involving specialties including ophthalmic surgeries in that intense light illumination could cause discomfort or even be harmful to the eye [5, 6].

The results demonstrated that the highly sensitive HD video camera can record high-quality images with good brightness and resolution under low light illumination. At present, many ophthalmic surgeries such as cataract, glaucoma, and others are performed under topical anesthesia. This means that the patient can experience the light level during the surgery, and bright light exposures can cause excessive dazzling and uncomfortable sensations. The 3D digital surgery is the key point to future advances to minimally invasive ophthalmic surgeries [7].

Retinal phototoxicity is a rare but proven risk of ocular surgery [5, 6]. Digital image enhancements help reduce the illumination to a considerable degree, decreasing the phototoxicity to the retinal pigmented epithelium cells and photoreceptors [8].

During anterior segment surgery, e.g., cataract and glaucoma, the surgical field is clearly visible even with the preset 2% and 6% main and retroillumination light sources. However, in five cases, we had to increase the retroillumination light source brightness to 7-8% (609–771 lux) of the maximum to improve the visibility during the phacoemulsification procedures. Importantly, none of the patients reported a dazzling sensitive and discomfort during the surgery.

In vitreoretinal surgery, remarkable technological advances have been made including the small gauge instruments and port systems. This has the advantage of a small wound size with few injuries to eye tissues. However, it has some disadvantages such as less flow of the infusion solution and restricts the illumination system due to small diameter of the light fiber pipe that forces the surgeon to use extremely bright light sources. In this study, all vitrectomies were performed with a 27-gauge port system, and the endoillumination level was initially set to 3% of the maximum output and gradually decreased to 2%. The wide-field spread chandelier light was set to 3%. Adam et al. reported that most surgeons feel comfortable when using endoillumination set at 10% with 3D HU surgery platform and the brightness was acceptable (TrueVision Visualization System, Santa Barbara, CA) [6]. In their study, the iris aperture of the camera was set at 80%, and in our study, we set the iris aperture at 60% to improve depth of focus. This means that less light from the operative field will reach the camera sensors and make it more difficult to obtain the good images at the camera output. In our small sample of cases, we found that in many cases, the endoillumination set at 3% was acceptable by the surgeon to perform vitrectomy.

In conclusion, the heads-up 3D system is a good alternative for minimizing the brightness of the illumination system. The system used in this study uses the latest technology on viewing objects at very low light exposure and shows that it can produce images 23 times brighter than that of the real images at the microscope's eyepieces. Also, it can keep the created images with a minimal loss in brightness in spite of reducing the aperture diameter of the iris. The role of the highly sensitive HD camera is to provide good depth of focus and also good quality in brightness and low level of noise during ophthalmic surgery with extremely low light exposure.

## Data Availability

The data used to support the findings of this study are available from the corresponding author upon request.
